# Synthesis of 1,1′-Bis(1-Methyl/Chloro-2,3,4,5-Tetraphenyl-1-Silacyclopentadienyl) [Ph_4_C_4_Si(Me/Cl)-(Me/Cl)SiC_4_Ph_4_] from Silole Anion [MeSiC_4_Ph_4_]^−^•[Li^+^ or Na^+^] and Silole Dianion [SiC_4_Ph_4_]^2−^•2[Li^+^]; Oxidative Coupling of Silole Anion [MeSiC_4_Ph_4_]^−^•[Li^+^ or Na^+^] by Ferrous Chloride (FeCl_2_) and Oxidative Coupling and Chlorination of Silole Dianion [SiC_4_Ph_4_]^2−^•2[Li^+^] by Cupric Chloride (CuCl_2_)

**DOI:** 10.3390/molecules24203772

**Published:** 2019-10-19

**Authors:** Jang-Hwan Hong

**Affiliations:** Department of Nanopolymer Material Engineering, Pai Chai University, 155-40 Baejae-ro (Doma-Dong), Seo-Gu, Daejon 302-735, Korea; jhong@pcu.ac.kr; Tel.: +82-42-520-5755

**Keywords:** silole, anion, dianion, oxidation, oxidative-chlorination, SET (single-electron transfer), cupric chloride, ferrous chloride

## Abstract

A reaction of silole anion {[MeSiC_4_Ph_4_]^−^•[Li^+^ or Na^+^] (**1**) with anhydrous ferrous chloride (FeCl_2_) in THF (tetrahydrofuran) gives 1,1′-bis(1-methyl-2,3,4,5-tetraphenyl-1-silacyclopentadienyl) [Ph_4_C_4_Si(Me)-(Me)SiC_4_Ph_4_] (**2**) with precipitation of iron metal in high yield. Silole dianion {[SiC_4_Ph_4_]^2−^•2[Li^+^] (**3**) is added to anhydrous cupric chloride (CuCl_2_) in THF at −78 °C, then the dark red solution changes into a greenish solution. From the solution, a green solid is isolated, and stirring it in toluene at room temperature provides quantitatively 1,1′-bis(1-chloro-2,3,4,5-tetraphenyl-1-silacyclopentadienyl) [Ph_4_C_4_Si(Cl)-(Cl)SiC_4_Ph_4_] (**4**) with precipitation of copper metal in toluene. The green solid is suggested to be 1,1′-bissilolyl bisradical [Ph_4_C_4_Si-SiC_4_Ph_4_]^2•^ (**8**), and lithium cuprous chloride salts {[Li_2_Cu^I^Cl_2_]^+^•[Cu^I^Cl_2_]^−^}. Both reactions are initiated by single-electron transfer (SET) from the electron-rich anionic silole substrates (**1** and **3**) to iron(II) and copper(II).

## 1. Introduction

Recent decades have witnessed tremendous progress in the field of group 14 metallole dianions and the related metallole anions [1,2,3,4,5,6] since the first silole dianion and germole dianion were reported [7,8]. In particular, the syntheses and characterizations of the silole anion [1-*tert*-butyl-SiC_4_Ph_4_]^1−^, the silole dianion [SiC_4_Ph_4_]^2−^, the bissilole dianion [Ph_4_C_4_Si-SiC_4_Ph_4_]^2−^, and the germole dianion [GeC_4_Ph_4_]^2−^ as aromatic compounds [8,9,10], previously considered merely as intangible intermediates, have been the starting point for exploring the synthetic, theoretical, and materials chemistry of group 14 metalloles and their anions [11,12,13,14,15,16,17,18,19,20,21,22,23,24,25,26,27], with increasing ASE (Aromatic Stabilization Energy) of the *η^5^*-lithium salts [C_4_H_4_E]^2−^•2[Li^+^] [16,17].

Very recently, conspicuous silole dianions {[SiC_2_(SiMe_3_)_2_C_2_Ph_2_]^2−^, SiC_2_(SiEt_3_)_2_C_2_Ph_2_]^2−^}, silole anion {[1-mesityl-SiC_2_(SiMe_3_)_2_C_2_Ph_2_]^1−^}, and bissilole dianion {[Ph_2_C_2_(Me_3_Si)_2_C_2_Si-SiC_2_(SiMe_3_)_2_C_2_Ph_2_]^2−^} were reported as having enhanced aromatic electronic structures, due to their trialkylsilyl groups on two α-carbons of the silole rings [28,29,30,31].

**Figure 1:**
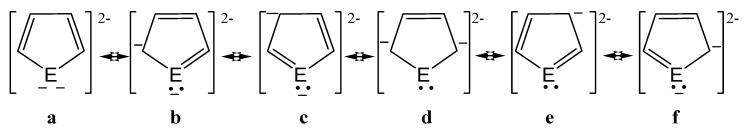
Mesomeric structures of group 14 metalloles dianions.

During the past three decades, all of the group 14 metallole and bismetallole dianions, from silicon to lead, have been explored as readily available highly aromatic systems [5,6], since the following mesomeric structures have had been suggested to denote their electronic characteristics in the pioneering works (Figure 1) [8,9,10].

Nowadays, even group 13 dilithio metalloles dianion and dilithio transition metalloles are prepared and characterized as aromatic dianion metalloles [32].

In general, reactions of silole anions and silole dianions with electrophiles are nucleophilic additions to E atoms of E–X bonds in the electrophiles, to make new Si–E bonds (E=C, Si, Ge, Sn, X=Cl, Br, I) [1,2,3,4,5]. In the case of the silole dianion, the respective reaction pathway depends on what the electrophile is—for example, dichlorocyclopropane, diphenylcyclopropenone, 2-adamantanone, 1,3-butadienes, and *N*,*N*′-di-*tert*-butylethylenediimine—due to the presence of two anionic electron pairs [33,34,35,36,37]. Nevertheless, all of them have been initiated by formal two-electron transfer.

Here, we report two oxidation reactions of silole anion [MeSiC_4_Ph_4_]^−^•[Li^+^ or Na^+^] with ferrous chloride (FeCl_2_) and silole dianion [SiC_4_Ph_4_]^2−^•2[Li^+^] (**1**) with cupric chloride (CuCl_2_). To date, there are no reports on the single-electron oxidation and/or chlorination of them, as far as we know. This is the first report for oxidative chlorination of silole dianion with cupric chloride (CuCl_2_) through a radical silole moiety, via two single-electron transfers.

## 2. Results

**Scheme 1:**
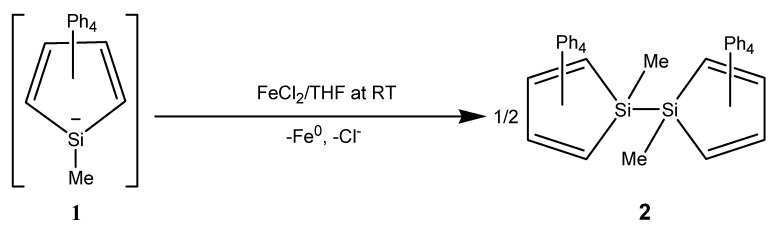
Reaction of silole anion [MeSiC_4_Ph_4_]^−^•[Li^+^ or Na^+^] (**1**) with FeCl_2_.

The addition of silole anion salt [MeSiC_4_Ph_4_]^−^•[Li^+^ or Na^+^] (**1**) to pale brown anhydrous ferrous chloride (FeCl_2_) in THF with stirring, immediately changed a dark violet solution into a black solution, with precipitation of black particles at room temperature. From the reaction mixture, 1,1′-bis(1-methyl-2,3,4,5-tetraphenyl-1-silacyclopentadienyl) [Ph_4_C_4_Si(Me)-(Me)SiC_4_Ph_4_] (**2**) was obtained quantitatively, and the black particle was identified as iron metal by its strong para-magnetism in Scheme 1.

**Scheme 2:**
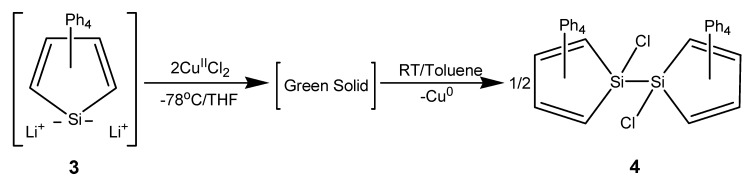
Reaction of silole dianion {[SiC_4_Ph_4_]^2−^•2[Li^+^] (**3**) with cupric chloride (CuCl_2_).

In another reaction, silole dianion {[SiC_4_Ph_4_]^2−^•2[Li^+^] (**3**)} was reacted with brownish anhydrous cupric chloride (CuCl_2_) in THF (tetrahydrofuran) at −78 °C, then immediately the dark red solution of the silole dianion (**3**) changed to a greenish solution. From the solution, a green solid was isolated quantitatively. ^1^H, ^13^C, or ^29^Si NMR spectroscopy was not applicable to the solid, due to its strong para-magnetism. The green solid was put into toluene, and stirring the solution at room temperature yielded 1,1′-bis(1-chloro-2,3,4,5-tetraphenyl-1-silacyclopentadienyl) [Ph_4_C_4_Si(Cl)-(Cl)SiC_4_Ph_4_] (**4**) quantitatively, with precipitation of copper metal on the surface of glassware (Scheme 2).

## 3. Discussion

**Scheme 3:**
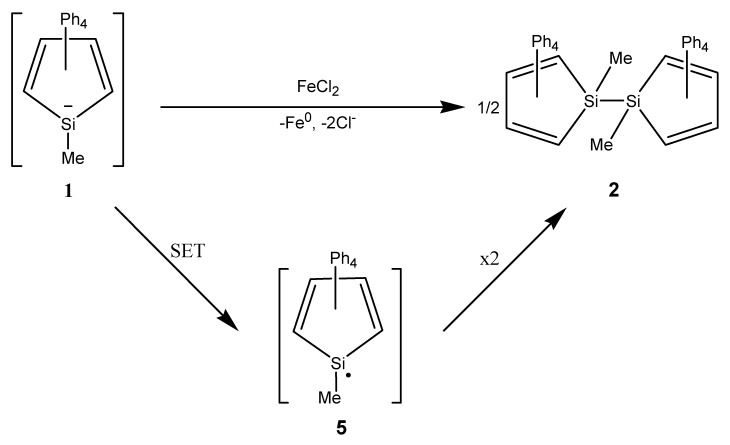
Generation of silolyl radical [MeSiC_4_Ph_4_]^•^ (**5**) via single-electron transfer (SET) and its dimerization.

Oxidation of the silole anion [MeSiC_4_Ph_4_]^−^•[Li^+^ or Na^+^] (**1**) by ferrous chloride (FeCl_2_) at room temperature might generate the silolyl radical [MeSiC_4_Ph_4_]^•^ (**5**) via single-electron transfer (SET), as in Scheme 3. Then, the coupling of the silolyl radical (**5**) immediately gives the bissilole (**2**). Alternatively, the silole radical (**5**) is able to be generated and dimerized via an unstable iron complex of [Ph_4_C_4_Si(Me)-Fe-(Me)SiC_4_Ph_4_], or a reductive elimination of the bissiole (**2**) from the iron complex, with an elimination of iron (0).

Although similar coupling reactions to Wurtz coupling are known for reactions of 1-choro-1-Ph/Me-2,3,4,5-tetraphenyl-1-silacyclopentadienes {[Cl,Ph-SiC_4_Ph_4_] and [Cl,Me-SiC_4_Ph_4_]}, with one equivalent of lithium or sodium, to give the respective bissiloles {[Ph_4_C_4_Si(Ph)-(Ph)SiC_4_Ph_4_] and [Ph_4_C_4_Si(Me)-(Me)SiC_4_Ph_4_]} [25,38], it is uncertain whether those reactions proceed via two-electron transfer or one-electron transfer. Nevertheless, the formation of the bissilole [Ph_4_C_4_Si(Me)-(Me)SiC_4_Ph_4_] (**2**) in Scheme 3 provides evidence for the dimerization of the silolyl radical [MeSiC_4_Ph_4_]^•^ (**5**), or for the reductive elimination of the bissiole (**2**) with iron metal from an iron complex {[Ph_4_C_4_Si(Me)-Fe-(Me)SiC_4_Ph_4_], since the radical species of (**5**) might not be stabilized enough to be alone. However, from the same reaction at −78 °C, the oxidation reaction, such as the reaction at room temperature, was not observed, and it was not clear whether some kind of bond was formed between an iron atom and two silole moieties.

The reaction in Scheme 2 clearly shows the oxidation of the silole dianion (**3**) and chlorination of each silicon atom in a bissilole. Nevertheless, there are a couple of controversial issues surrounding the chemical bond formation of Si–Cu as an intermediate in the change of oxidation state for copper and reaction mechanism, since most of the mechanisms for copper-catalyzed organic reactions are still uncertain and controversial [39].

For more than a century, copper has been used to promote cross-coupling reactions of aryl halides with diverse nucleophiles to construct C–C and C–heteroatom bonds in organic synthesis [40]. Oxidative couplings of carboanion species, such as Grignard reagents and organo-lithium, via copper organyls with oxygen or other oxidants, are well-known reactions for organic synthesis [41,42]. Numerous successful Cu-catalyzed aerobic oxidation reactions, in which the use of Cu(II) species obviates the introduction of an additional oxidizing agent, have been developed for fine chemical, pharmaceutical, and related industries [41,42,43,44,45]. Although the mechanisms of those reactions, including Ullmann-type reactions, are not clearly known, in recent years those reactions are classified into three general mechanistic pathways, depending on organic substrates [39]. The first step in the most well-known mechanism among three mechanistic pathways is initiated by single-electron transfer (SET) from electron-rich substrates, such as 1,3,5-trimethoxy benzene, to Cu(II), which is reduced to Cu(I). Then, chlorination of the oxidated cationic radical of 1,3,5-trimethoxy benzene gives 2-chloro-1,3,5-trimethoxy benzene in high selectivity [25]. The high selectivity of the oxidative chlorination is attributed to its deactivation of the product 2-chloro-1,3,5-trimethoxy benzene to the Cu-catalyst, due to the electron-withdrawing effect of the substituted chlorine atom. Neutral arenes, such as benzene, are unreactive under these conditions [46,47,48,49,50,51].

**Scheme 4:**
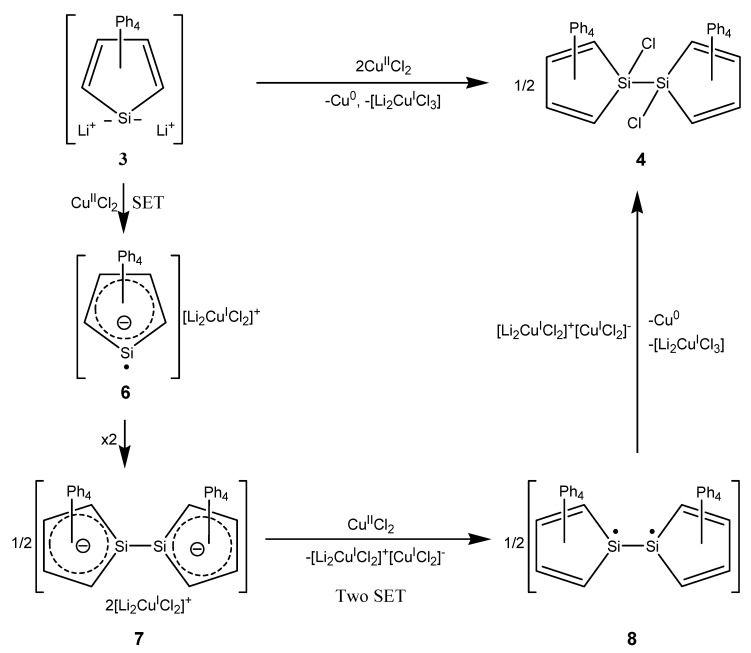
Oxidative chlorination mechanism of the silole dianion {[SiC_4_Ph_4_]^2−^•2[Li^+^]} (**3**) by cupric chloride (CuCl_2_), via single-electron transfer and chlorination on each silicon atom of the silole moiety.

At these points, the silole dianion {[SiC_4_Ph_4_]^2−^•2[Li^+^]} (**3**) is unquestionably a real, electron-rich aromatic substrate. Therefore, the silole dianion (**3**) is readily oxidized to a silole radical anion cuprate [SiC_4_Ph_4_]^•−^•2[Li_2_Cu^I^Cl_2_]^+^ (**6**) by cupric chloride (CuCl_2_) at −78 °C, via single-electron transfer (SET), in Scheme 4. Then the silole anion radical [SiC_4_Ph_4_]^•−^ (**6**) might be immediately dimerized to the bissilole dianion cuprate {[Ph_4_C_4_Si-SiC_4_Ph_4_]^2−^•2[Li_2_Cu^I^Cl_2_]^+^} (**7**). Several bissilole dianions, including the same bissilole dianion {[Ph_4_C_4_Si-SiC_4_Ph_4_]^2−^•2[Li]^+^} [10], {[Me_4_C_4_Si-SiC_4_Me_4_]^2−^•2[K^+^]} [12], {[Ph_2_C_2_Me_2_C_2_Si-SiC_2_Me_2_C_2_Ph_2_]^2−^•2[Na^+^]} [52], and {[Ph_2_C_2_(Me_3_Si)_2_C_2_Si-SiC_2_(SiMe_3_)_2_C_2_Ph_2_]^2−^•2[Na^+^](DME)} [31], have been reported and characterized as stable aromatic systems. In addition, air oxidation of 1-silafluorenyl dianion has been reported to give 1,1′-bis(silafluorenyl) dianion, besides germanium analogue of the dianion [53,54].

The bissilole dianion (**7**) is still an electron-rich aromatic substrate to cupric chloride (CuCl_2_) in Scheme 4. Therefore, two single-electron transfers from the bissilole dianion (**7**) to two Cu(II) readily gives 1,1′-bis(radical silolyl) [Ph_4_C_4_Si-SiC_4_Ph_4_]^2•^ (**8**) with two equivalents of cuprous dichloride anion {[Cu^I^Cl_2_]^−^}, since there is not enough space for four α-phenyls around two silicon atoms in (**7**) to form 1,1′-disila-fulvalene in plane. The observed strong para-magnetism of the green solid is thought to be due to the radicals in (**8**). A stable free radical of the similar 2,3,4,5-tetraphenyl-silolyl species has also been reported [33]. Stirring the isolated green solid, the bisradical species (**8**) and lithium cuprous chloride salts of 2{[Li_2_Cu^I^Cl_2_]^+^•[Cu^I^Cl_2_]^−^}, in nonpolar toluene at room temperature, provides 1,1′-bis(1-chloro-silole) [Ph_4_C_4_Si(Cl)-(Cl)SiC_4_Ph_4_] (**4**) with strong Si–Cl bonds via chlorinations on each silicon atom of the bisradical species (**8**). There, a copper metal and active chlorine radical (or chlorine molecule) are generated from the cuprous dichloride anion {[Cu^I^Cl_2_]^−^} while the other chloride anion remains, then the active chlorine radical is coupled with the radical on each silicon atom of the bis(radical silolyl) (**8**) in Scheme 4. The coupling of the radical moiety (**8**) and the chlorine radical is consistent with other oxidative halogenation mechanisms of arenes, since the cationic radical intermediate of arene transforms into a neutral arene radical through its deprotonation step during halogenations [46,47,48,49,50,51].

In the above mechanism in Scheme 4, it is of interest that one copper(II) atom is reduced to copper metal by two single-electron transfers via Cu(I), while the other copper(II) atom is reduced into Cu(I) via single-electron transfer. Furthermore, among four equivalent chlorine atoms, one chlorine atom is consumed by the chlorination of the silicon atom in each siloyl moiety, and the other three chlorine atoms remain as chloride anions (2LiCl and Cu^I^Cl), since there is no chlorine source other than two equivalent cupric chloride (Cu^II^Cl_2_) in reaction Scheme 4. In general, oxychlorinations of arenes proceed with the copper catalyst in the presence of a chlorine source (LiX) [48,55,56,57].

In modern chemistry, triorganosilylcopper compounds are important reagents and the use of them in organic synthesis was pioneered by Fleming [57]. They are usually prepared in situ and can be used for the functionalization of alkenes and other reactions under mild conditions [45]. Generally, information on the structure of the silylcopper compounds, in general type Li[Cu(SiR_3_)_2_], is very limited even though such species could have some significance in the important Müller–Rochow industrial process, and there is still plenty of room for further development of copper-catalyzed silylations to make carbon–silicon bonds [58]. Although only couples of bulky silyl cuprates have been isolated and characterized, they have diverse structures such as ligand-stabilized silyl cuprate [Ph_3_SiCu(PMe_3_)_3_] [59], µ^2^-bridged hypersilyl cuprates [Li(THF)_3_][Cu_2_{Si(SiMe_3_)_3_}_2_Br] [60], Li[Cu_2_{Si(SiMe_3_)_3_}_3_] and Li[Cu{Si(SiMe_3_)_3_}_2_] [61], discrete linear structures of disilylcuprates Na[Cu{Si(*t*-Bu)_3_}_2_][THF]_2,4_ [62] and [K or Na][Cu_2_{Si(SiMe_3_)_3_}_3_] [63], and a nearly planar cyclic trimer of [Cu_3_{Si(SiMe_3_)_3_}_3_], with almost linear Si–Cu–Si in the solid state [64]. In particular, µ^2^-bridged di(hypersilyl)cuprates [Li(thf)_4_] [Cu{Si(SiMe_3_)_3_}_2_(CuCl)_4_] [65], which is synthesized from the reaction of copper(I) chloride with lithium hypersilanide at −78 °C in THF, is reported as an extremely kinetically unstable intermediate to decomposing above −30 °C, with precipitation of copper metal [65]. The precipitation of copper metal from copper(I) coincides with the suggestion of the mechanism in Scheme 4.

Nevertheless, this does not completely rule out the possibility that the isolate green solid is the bissilole dianion cuprate (**7**) containing unreacted cupric chloride (Cu^II^Cl_2_), since the observed paramagnetism might be due to the copper(II) with d^9^ electronic structure. There is another possibility, that the disproportionation of two equivalents of cuprous chloride (Cu^I^Cl), reduced from cupric chlorides (Cu^II^Cl_2_) via single-electron-transfer, provides copper metal (Cu^0^) and cupric chlorides (Cu^II^Cl_2_), which is then reduced to cuprous chloride (Cu^I^Cl), generating a chlorine radical, which is used for the chlorination of the silicon atom in each silolyl moiety. In addition, a ligand exchange reaction between the silole dianion (**3**) and chloride anion of cupric chloride (Cu^II^Cl_2_) could afford a disilene of 1,1′-disila-fulvalene, via a silolyl cuprates {(Silolyl)_2_Cu_2_}. Then, the disilene could react with chlorine to afford the chlorinated bissilole (**4**).

Determining which structure is the active intermediate (**7** or **8**) is of interest for future work surrounding other oxidation reactions.

## 4. Materials and Methods

All reactions were performed under an inert nitrogen atmosphere, using standard Schlenk techniques. Air-sensitive reagents were transferred in a nitrogen-filled glovebox. THF and ether were distilled from sodium diphenylketyl under nitrogen. Toluene was stirred over concentrated H_2_SO_4_ and distilled from CaH_2_. Melting points were measured with Wagner & Müntz Co. (München, Germany), Capillary Type. To dry the metal chloride, hydrous cupric chloride with an excess of chlorotrimethylsilane in THF was refluxed, with stirring, under nitrogen atmosphere for several hours [66].

1,1′-Bis(1-Me-2,3,4,5-tetraphenyl-1-silacyclopentadienyl) [Ph_4_C_4_Si(Me)-(Me)SiC_4_Ph_4_] (**2**): The silole anion {[MeSiC_4_Ph_4_]^−^•[Li^+^] or [MeSiC_4_Ph_4_]^−^•[Na^+^]} (**1**) was prepared according to the literature and used in situ [25]. Addition of silole anion salt (1.58 g, 3.96 mmol for the lithium salt, 1.61 g, 3.96 mmol for the sodium salt) to pale brown anhydrous ferrous chloride (0.29 g, 2.29 mmol, FeCl_2_) in THF changed the dark violet solution into a black solution, with precipitation of black particles. After completing the addition, it was stirred for 2 h. Workup of the mixture with distilled water and dichloromethane gave a pale green, unclear solution and black precipitation. Filtration of the mixture gave a green solution, from which removal of dichloromethane under reduced pressure gave a pale green solid. The solid was washed with ether for purification and identified as the bissilole (**2**) by the comparison of its analytical data with an authentic sample [25]. The back particle was identified as iron metal by its strong para-magnetism. Yield: 0.67 g (85%).

1,1′-Bis(1-chloro-2,3,4,5-tetraphenyl-1-silacyclopentadienyl) [Ph_4_C_4_Si(Cl)-(Cl)SiC_4_Ph_4_] (**4**): The silole dianion salt {[SiC_4_Ph_4_]^2−^•2[Li^+^]} (**3**) was prepared according to the literature used in situ [10]. The silole dianion solution (1.79 mmol) in THF was added to brownish anhydrous cupric chloride (CuCl_2_, 0.481 g, 3.58 mmol), with stirring, at −78 °C, and it was stirred for 3 h. Then a greenish solution was obtained. After the greenish solution was filtered and THF removed from the resultant, a green solid was isolated. The solid was dissolved in toluene, and the toluene solution was stirred for 1 h at room temperature. Then, metallic copper was precipitated on the surface of the reaction flask. Filtration of the mixture gave toluene solution, and the solution was concentrated under reduced pressure. The resultant solution was refrigerated for a couple of days and yellow crystals of (**4**) were obtained. Yield: 0.639 g (85%); mp 299 °C (Lit. 300–304 °C), Anal. Found: C 79.88, H 4.88, C_56_H_40_Cl_2_Si_2_ calcd.: C 80.07, H 4.80; ^29^Si-NMR (CDCl_3_, ref; ext. TMS = 0.00), 0.24 (Si); MS (M^+^, relative abundance) *m*/*z* 838, 419 (lit. [67]).

## 5. Conclusions

Silole anion [MeSiC_4_Ph_4_]^−^•[Li^+^ or Na^+^] (**1**) was oxidized by anhydrous ferrous chloride (FeCl_2_) at room temperature; 1,1′-bis(1-methyl-2,3,4,5-tetraphenyl-1-silacyclopentadienyl) (**2**) was obtained quantitatively with reduced iron metal. From the reaction of the silole dianion [SiC_4_Ph_4_]^2−^•2[Li^+^] (**3**) with cupric chloride (CuCl_2_) at −78 °C in THF, a green solid was isolated. The green solid in toluene was transformed at room temperature into 1,1′-bis(1-chloro-2,3,4,5-tetraphenyl-1-silacyclopentadienyl) (**4**) quantitatively, with precipitation of copper metal on the surface of the reaction flask.
